# Post Partum Death in a Patient Diagnosed With COVID-19

**DOI:** 10.3389/fgwh.2020.567810

**Published:** 2020-09-24

**Authors:** Atanas Sivevski, Dafina Karadzova, Natasha Davceva, Irena Aleksioska-Papestiev, Romir Kadriu, Ivan Velickovic, Ivana Markovic, Nada Pejcic, Curtis L. Baysinger

**Affiliations:** ^1^Department of Anesthesiology, University Hospital of Gynecology and Obstetrics, Saints Cyril and Methodius University of Skopje, Skopje, North Macedonia; ^2^Institute of Forensic Medicine, Criminology, and Medical Deonthology, Saints Cyril and Methodius University of Skopje, Skopje, North Macedonia; ^3^Department of Gynecology and Obstetrics, University Hospital of Gynecology and Obstetrics, Saints Cyril and Methodius University of Skopje, Skopje, North Macedonia; ^4^University Clinic for Infectious Diseases and Febrile Conditions, Saints Cyril and Methodius University of Skopje, Skopje, North Macedonia; ^5^Department of Anesthesiology, SUNY Downstate Health Sciences University, Brooklyn, NY, United States; ^6^Department of Radiology and Molecular Imaging Sultan Qaboos University Hospital, Muscat, Oman; ^7^Department of Anesthesiology, Leskovac General Hospital, Leskovac, Serbia; ^8^Department of Anesthesiology, Vanderbilt University Medical Center, Nashville, TN, United States

**Keywords:** corona virus disease (COVID-19), maternal mortality, respiratory failure, infectious disease, vertical transmission

## Abstract

**Background:** There are few case reports describing maternal mortality and intensive care of the pregnant patient with COVID-19 infection.

**Case:** A 27-year-old patient at 34 weeks of gestation was admitted for the evaluation of cough, fever, tachypnea, and oligohydramnios. The day of admission she underwent cesarean delivery for a non-reassuring fetal heart rate tracing. Over the next 6 days her clinical condition deteriorated, she developed multi organ system failure, and died despite aggressive supportive care.

**Conclusion:** Although mortality related to COVID-19 in pregnancy has been rarely reported to date, we describe a case of progressive clinical deterioration postpartum despite aggressive supportive care. Management strategies specific for pregnant women have not been developed. In timing delivery, the obstetrician must consider the possibility that the inflammatory response associated with CD may increase the risk for multiorgan system failure in parturients with COVID-19 while recognizing that risks to the fetus may be higher in patients with COVID-19 than in other critically ill parturients. Vertical transmission of infection to the neonate did not occur in our case and has not been demonstrated in other pregnancies with COVID-19 disease.

## Introduction

Corona virus disease has evolved into the worst worldwide pandemic since the influenza outbreak of 1918 (1). Although case series describe the disease course in pregnant women (2–5), there are few published reports of maternal death (6–8). We report such a case that occurred in Skopje, Macedonia in a woman who was symptomatic for COVID-19 at the time of delivery, became progressively more ill over the next 6 days, and died despite aggressive supportive care. The patient's family provided consent for publication of this report and accompanying images.

## Case

A 27-year-old G2P1 woman at 34 weeks gestation was admitted with a 4-day history of sore throat, cough, fever, and shortness of breath at the University Hospital of Gynecology and Obstetrics, Skopje, North Macedonia. Her past medical and obstetrical history was unremarkable, and her current pregnancy had been without incident until oligohydramnios was discovered on a routine screening ultrasound examination the day before admission. Her social history revealed that a father in law who resided with her was positive for COVID-19, and she resided in a community where COVID-19 was widespread.

**Figure 1:**
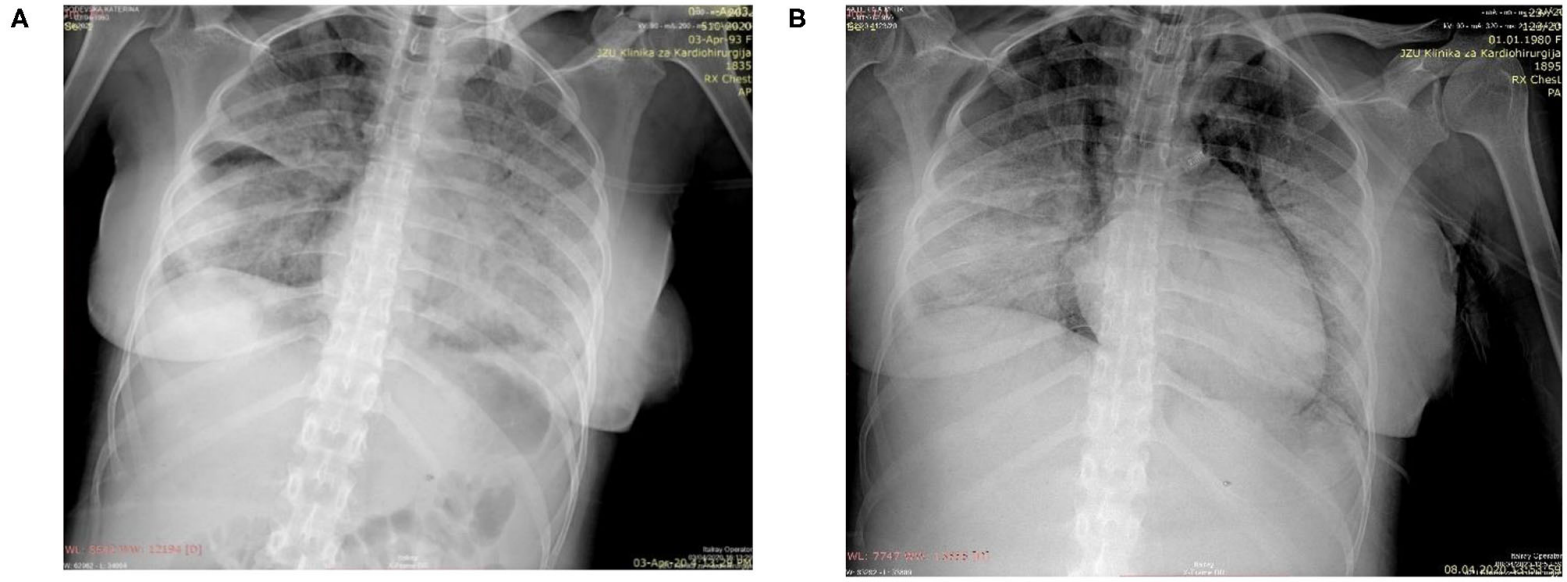
**(A)** Chest radiograph on day of delivery showing extensive diffuse bilateral opacities suggestive of pneumonia; **(B)** Chest radiograph taken following endotracheal intubation on the 6th post-delivery day showing progression of pnuemonia, subcutaneous emphysema, pneumomediastinum, and prominent right heart contours suggestive of right heart failure.

Physical examination revealed a slightly dyspneic and febrile woman with a BP 115/70 mmHg, pulse 80 beats per minute, respiratory rate of 32 breaths per minute, an oxygen saturation of 94% on room air, temperature 38.1°C, and a BMI of 23.4 kg/m^2^. Although her pulmonary examination was clear to auscultation, she could not hold her breath for >3 s. Initial laboratory findings (Table 1) showed a lymphopenia and no other significant abnormality. Her chest radiograph on the day of admission showed severe bilateral coalescent consolidative opacities suggestive of pneumonia (Figure 1A). Given the patient's presentation, naso, and oropharyngeal swabs were obtained for SARS-CoV-2RT-PCR test. She was placed in isolation and contact precautions were initiated using World Health Organization (WHO) guidelines (7). The test was reported positive 12 h later using WHO procedures for quantitative RT-PCR testing. An initial fetal evaluation with trans-vaginal and abdominal ultrasound and a non-stress test were reassuring with normal fetal heart rate with minimal variability and no uterine contractions. Fetal biophysical measurements were normal for gestational age with an amniotic fluid index of 2. A vaginal examination showed a cervix that was long thick and closed, and her Bishop's score was 2.

**Table 1:** Laboratory results during the patient's hospital stay.

**Blood Laboratory test**	**Day of admission/delivery**	**4 Days after delivery**	**5 Days after delivery**	**6 Days after delivery**
Hemoglobin, gr/dL	11.0	12.1	12.0	9.8
WBC × 10^9^/L	7.2	15.0	17.1	19.3
Lymphocytes, %	7	7	6	10
Neutrophils, %	89	89	91	84
Platelets × K/micL	249	231	208	306
Creatinine, md/dL	0.46	0.45	0.34	2.73
BUN, mg/dL	11	31	33	83
Sodium, mEq/L	140	146	142	144
Potassium, mEq/L	3.9	3.4	3.3	5.8
Calcium, mEq/L	1.97	2.08	1.93	1.73
AST, U/L	24	12	18	42
ALT, U/L	55	62	46	251
LDH, U/L	764	1,622	1,464	2,022
D-Dimer, ug/mL			35,712	
CPK, U/L	123	131	242	1,341
CK-MB, U/L				38
Troponin, ng/mL				7.73
PT, seconds			12.8	
PTT, seconds			36.4	
**Arterial blood gas**				
pH			7.52	
pCO_2_, mmHg			25.2	
pO_2_, mmHg			57.8	
BE			−0.5	
O_2_ saturation, %			91	

*AST, aspartate aminotransferase; ALT, alanine aminotransferase; CPK, creatine phosphokinase; CK-MB, creatine kinase-MB; PT, prothrombin time; PTT, partial thromboplastin time; BE, base excess*.

The patient was evaluated by the obstetric, infectious disease, and anesthesia services and a multidisciplinary plan was made. The patient received initial oxygen therapy with nasal cannula to maintain an oxygen saturation > 95%. A biophysical profile performed 12 h after admission showed a score of 4 and a dose of dexamethasone was administered. An induction of labor with oxytocin was stopped after severe late decelerations were noted. Because of the positive oxytocin challenge test and the potential for rapid maternal respiratory deterioration, the decision was made to proceed with an urgent cesarean delivery (CD) on the day of admission.

After antibiotic prophylaxis with 2 gm ceftriaxone, a successful CD was performed under spinal anesthesia with 10 mg isobaric 0.5% bupivacaine with 20 mcg fentanyl and 100 mcg morphine. All personnel followed WHO guidelines for isolation during the procedure. A vigorous 2.15 kg male with Apgar's 8 and 8 was kept in isolation after delivery and throat swabs for SARS-CoV-2RT were obtained. Postoperatively, the mother was returned to isolation in the recovery area. On the third day following delivery, the neonate's SARS-CoV-2RT test was reported negative and he was released from isolation.

After adequate recovery from anesthesia and assurance of no obstetrical complications, the mother was transferred the first post-delivery day to the University Clinic of Infectious Diseases hospital for further monitoring and treatment. Over the next 4 days, the patient was persistently febrile, with peek temperatures of 39°C, developed increasing shortness of breath despite antimicrobial therapy with meropenem, bronchodilator therapy with aminophylline, intravenous fluid, and anticoagulant therapy with therapeutic enoxaparin. A non-rebreathing mask was placed on the 4th post-delivery day. On the 5th day, she became somnolent, tachypneic (respiratory rate >40), her systolic BP was consistently below 90 mmHg, and her oxygen saturations fell to 80% despite oxygen flow rates > 10 L/m per non-rebreathing mask. An arterial blood gas showed a respiratory alkalosis and moderate hypoxemia. Her laboratory results showed a normal partial thromboplastin and prothrombin times despite a significantly elevated D-Dimers. (Table 1) She was endotracheally intubated, transferred to the intensive care unit, sedated, and vasopressor therapy with norepinephrine at 2.6 mcg/min and dobutamine at 500 mcg/min were begun. Her oxygenation saturation improved to > 95% after intubation with a peak inspiratory pressure of 40 cm H_2_O, minute ventilation of 7 L/min, an FIO_2_ of 100%, and PEEP of 12 cm H_2_O; her hemodynamics improved. However, over the next 24 h she became increasingly hypoxic (oxygen saturations <80%) despite adjustments in ventilator settings. On the morning of the 6th post-delivery day, she showed laboratory signs of renal insufficiency, liver dysfunction, and significant myocardial damage (Table 1). She had a cardio-respiratory arrest later that evening from which she could not be resuscitated. A repeated chest radiograph taken just before her arrest showed progression of respiratory disease complicated by pneumomediastinum and right heart failure (Figure 1B).

## Discussion

We describe a maternal death following delivery in a pregnant patient suffering from COVID-19. Our patient suffered a cardiopulmonary arrest due to multi-organ system failure with significant signs of heart failure and myocardial damage. Her final radiograph showed evidence of pneumomediastinum, a sign of alveolar sac compromise, which could have abruptly progressed to a tension pneumothorax. Tension pneumothorax occurs in 30–60% of patients who are mechanically ventilated in the setting of multi-organ system failure (9). Spontaneous pneumothorax has been recently described in non-pregnant patients with CVOID-19 who present with respiratory distress (10). Although tension pneumothorax often leads to abrupt hemodynamic failure, our patient's rapid decline in respiratory and cardiac function over the day and a half prior to her arrest was most likely irreversible.

Hantoushzadeh et al. has reported the largest and most detailed case series of maternal deaths (8). Our patient presented with symptoms of dyspnea, fever, cough, and lymphopenia similar to the signs and symptoms reported in their case series and other case reports (2–8). Her course following delivery was also similar with increasing signs of respiratory failure and subsequent cardio-pulmonary failure despite maximal supportive therapy. In our patient, like others, the multi-organ failure that frequently accompanies ARDS was most likely causative, although the relative contributions of respiratory failure, pulmonary thromboembolism, and heart failure cannot be determined.

It is difficult to determine an accurate number of pregnant women who had COVID-19 infection, as many case series and systematic reviews include the same patients (6–8). Reports citing numbers of infections among pregnant patients are inaccurate as many reports come from areas in which undercounting of persons with infection is likely. Data from Centers for Disease Control in the U.S. suggest an overall death rate of 0.4% among persons 20–45, with women ~2/3 less likely to die than men (11).

The physiological adaptations of pregnancy are thought to predispose parturients to greater risk for pulmonary and cardiac decompensation and a more severe course during pulmonary infection (12). Also, changes in the maternal immune response during pregnancy are thought to increase the risk of pulmonary infection from viruses other than COVID-19 and from bacterial pneumonias (12). However, an overly vigorous immune response may significantly contribute to the syndrome of respiratory and multi-organ failure reported in younger patients. The reduction in interleukin and cytokine release that occurs in normal pregnancy may reduce this response and thus decrease the risk for death in infected pregnant women (13). Although the absence of maternal death in early observational studies of COVID-19 suggests that pregnancy may protect against mortality, previous case series and our case report reinforce that this risk is not zero (1–5, 13–15). In the study by Hantoushzadeh et al., none of the household members of infected women died. In our case, the patient's father in law died from COVID-19. Surveillance studies with long term follow up that account for differences in baseline maternal mortality and adjust for potential co-morbidities that affect risk have not been done.

COVID-19 patients should not be delivered based on infection alone, but for obstetric or fetal indications (15). Timing delivery in the critically patient with COVID-19 may be difficult. Our patient was at risk for respiratory decompensation when admitted, showed signs of fetal non-well-being, and we chose to deliver her by urgent, non-emergent CD at 34 weeks. Many critically ill patients may be successfully cared for periods of time prior to delivery with good outcomes (16) and CD, like all surgical procedures, increases the maternal systemic inflammatory response (17). This increase may add to the overly vigorous innate immune response that is detrimental in patients with COVID-19, although as noted above, the attenuated immune responses that accompany pregnancy may be protective. Risks to the fetus in timing delivery must be carefully considered. Neonatal mortality may be higher in COVID-19 patients than among other critically ill parturients. Six of 11 neonates in the case series by Hantoushzadeh et al. died *in utero* or shortly after birth, despite the descriptions of good fetal surveillance (8).

Stroke and embolic and thrombotic disease of other organs systems is thought to significantly contribute to death from COVID-19 in younger victims, and some authors suggest that pregnant women may be no different (18). It is possible that thromboembolism significantly contributed to our patient demise. Her chest radiograph just before her cardiopulmonary arrest suggested right heart failure which often accompanies pulmonary thromboembolism and had markedly elevated d-Dimers just prior to death; however, significant elevation of d-Dimers often occurs from the disseminated intravascular coagulation associated with ARDS.

Cesarean delivery increases the risk for maternal thromboembolic events when compared to vaginal delivery (19) and the risk for thromboembolism after CD might be accentuated in parturients with COVID-19. If it is a significant contributor, then the routine post CD prophylactic measures described in all previous reports may not prevent it.

Like most of the pregnant women with COVID-19, our patient delivered preterm (2–8). The baby did not test positive for COVID-19 and did not exhibit any symptoms. This is similar to the report by Hantoushzadeh et al. which showed no vertical transmission in the 4 neonates who were tested (8). In those few cases where it has been suspected the neonate may have been infected following delivery (13). The virus has not been detected in amniotic fluid, umbilical cord blood, and is rarely present in the naso-pharyngeal tract of infants whose mothers had COVID-19 (13).

## Conclusion

We present a case of death due to COVID-19 in a pregnant woman following delivery. Although maternal mortality following delivery has been infrequently reported and early surveys of patient outcomes suggest that the death rate among pregnant women with COVID-19 is different than among non-pregnant women, this conclusion nay be inaccurate. Thromboembolism may contribute to the respiratory failure accompanying COVID-19, but whether this risk is altered by pregnancy is unknown. In timing delivery, the obstetrician must consider the possibility that the inflammatory response associated with CD may increase the risk for multiorgan system failure in parturients with COVID-19 while recognizing that risks to the fetus may be higher in patients with COVID-19 than in other critically ill parturients. Post-delivery supportive care is like that in non-pregnant women and strategies specific for the pregnant women have not been developed. Vertical transmission of COVID-19 has not been conclusively demonstrated.

## Ethics Statement

The Institutional Review Board of the University Hospital of Gynecology and Obstetrics, Skopje, North Macedonia provided permission for publication of this report and images as the deceased patient's family was lost to follow-up.

## Author Contributions

CB, IV, IM, and NP assisted in the writing of the manuscript and concurs with its content. AS, DK, ND, IA-P, and RK participated in care of the patient and in the writing of the manuscript and concurs with its content. All authors contributed to the article and approved the submitted version.

## Conflict of Interest

The authors declare that the research was conducted in the absence of any commercial or financial relationships that could be construed as a potential conflict of interest.
